# Effects of Agility Training with a Light-Based System on Balance and Functional Performance in Individuals with Parkinson’s Disease

**DOI:** 10.3390/healthcare13202559

**Published:** 2025-10-11

**Authors:** Thelma Rut Holmarsdottir, Andri Thor Sigurgeirsson, Atli Agustsson

**Affiliations:** Research Centre of Rehabilitation and Movement Science, Department of Physical Therapy, Faculty of Medicine, University of Iceland, 102 Reykjavík, Iceland

**Keywords:** Parkinson’s disease, agility training, motivation, postural balance

## Abstract

**Background/Objectives:** Impaired balance and general mobility are common complications of Parkinson‘s disease (PD) and are largely caused by bradykinesia and hypokinesia. Although previous studies have shown that patients can increase the speed and amplitude of movement with training, apathy, which is also common among people with PD, reduces this prospect. Training with light pods was originally developed for athletes to enhance agility in a way that is motivating. However, this type of training could be ideal for individuals with PD and possibly reduce bradykinesia and its effects. This study used a longitudinal interventional design without a control group to explore the effects of a four-week agility training with light equipment on balance and general mobility in patients with PD, as well as to assess motivational properties. **Methods**: Seven individuals with PD of the motor subtype “akinetic–rigid” participated in this study. Each participant received training three times per week for four weeks. The training session consisted of five rounds; in each round, participants had to turn off 20 lights. Measurements were performed one and a half weeks before training, at the beginning of the program, and at the end of the program. Balance was assessed with Mini-BESTest, general mobility with Timed Up and Go (TUG), transfer skills with 5× Sit to Stand, walking speed with the 10 m walking test, and the ability to turn on a spot with the 360° Turn Test. Motivational aspects of training were assessed after each training session, with scoring on a scale of 0–10. **Results**: The training significantly improved overall balance (*p* < 0.001), especially reactive postural control, sensory orientation, and dynamic gait, while anticipatory balance remained unchanged. Turning ability improved, but mobility, transfer ability, and walking speed did not. Motivation remained consistently high across participants. **Conclusions**: A four-week light-based agility training program can improve balance and turning ability in people with PD and appears to be motivating. However, no clear effects were found for general mobility, transfer skills, or walking speed. Given the small sample size and absence of a control group, these findings should be interpreted with caution.

## 1. Introduction

Parkinson’s disease (PD) is one of the most common neurodegenerative disorders, predominantly affecting older individuals [1,2]. It is characterized by the degeneration of dopaminergic neurons in the substantia nigra, leading to impairments across motor, cognitive, and affective domains [3]. Motor symptoms are most evident as hypokinesia and bradykinesia [4,5], particularly in the akinetic–rigid subtype [6], affecting gait, balance, turning, and sit-to-stand tasks.

These impairments are further exacerbated during dual-tasking and compounded by non-motor symptoms such as depression, apathy, and fatigue [7,8,9].

Physical exercise and targeted training have consistently shown benefits for motor performance [10,11,12,13,14] and fall prevention in PD, supporting neuroplasticity despite slower motor learning [13,15,16] in PD; this can strengthen existing dopaminergic pathways and help form new non-dopaminergic connections.

Over the past four decades, numerous physical therapy modalities have proven effective in addressing PD-related disabilities [17]. Step training, especially when focused on recalibrating hypokinetic movements, has shown promising effects on both reactive and anticipatory balance [16,18,19,20,21,22]. However, agility training, designed to enhance rapid, multidirectional movements, has been rarely studied in PD, despite bradykinesia being a hallmark feature.

Light-based training systems, originally developed for athletes, may provide an innovative and motivating approach for individuals with PD. These systems combine agility and cognitive challenges, deliver immediate feedback, and facilitate flexible customization of training load [23,24]. Such features could make them particularly suitable for improving balance and promoting adherence in PD rehabilitation.

Therefore, the aims of this study were as follows:

- To assess whether light-based agility training improves balance in people with Parkinson’s disease and which aspects of balance are affected.
- To examine effects on functional performance, specifically, transfer ability, walking speed, and turning.
- To evaluate how motivated participants view this type of training and whether motivation changes over time.

## 2. Materials and Methods

This study used a longitudinal intervention design without a control group with a small sample. These limitations restrict generalizability and prevent ruling out learning or medication effects, but the design reflected practical constraints related to participant availability, time, and resources. Measurements were taken one and a half weeks before, immediately before, and immediately after the intervention. All participants received the intervention. The study protocol was approved by the Icelandic National Bioethics Committee (VSN-21-131).

Participants were recruited through neurologists and had to be diagnosed with Parkinson’s disease, specifically the “akinetic–rigid” subtype. Inclusion criteria were based on the Unified Parkinson’s Disease Rating Scale (UPDRS) [25] and Hoehn and Yahr stages II–III [26]; see Table 1.

Participants could not have other conditions affecting gait or balance, nor a history of brain surgery. They had to attend at least 8 out of 12 training sessions to be included in the study. Medication was to remain unchanged from three weeks before to the end of the study. Although not verified, participants were told to keep their physical activity levels unchanged, as the intervention was meant to be an addition.

Baseline physical activity levels were evaluated using the Saltin–Grimby Physical Activity Level Scale (SGPALS [27]). Balance was measured with the Mini-BESTest [28], which has good predictive value for fall risk [29]. General mobility was assessed using the Timed Up and Go (TUG) test [30]. Transfer ability was evaluated with the 5× Sit to Stand test [31]. Gait speed was measured with the 10 m walk test [32]. Turning ability was assessed using the 360° Turn Test, which is reliable for individuals with Parkinson’s disease [33]. After each training session, motivation was rated on a scale from 0 (not motivating at all) to 10 (highly motivating).

Training was conducted three times per week for four weeks at the University of Iceland using the BlazePod^®^ light system (BlazePod Ltd., Tel Aviv, Israel). This system, shown to be reliable in athletes, has not been evaluated for people with Parkinson’s disease [34]. Sessions occurred in the late afternoon at a consistent time.

Six BlazePod^®^ lights were placed in a 250 cm diameter circle on 50 cm risers, with a cross marked in the center for participant positioning. Participants, barefoot, tapped each light with their hand as it lit up. Each session consisted of five sets of 20 lights. A one-minute rest was provided between sets to prevent effort from being affected by fatigue. An app recorded total and average reaction times. Participants followed standardized verbal instructions before and after sets. Included in the instruction before the first set was the following line: “Try to take big steps toward the light that turns on and be quick to figure out the next light”.

Data was prepared and analyzed using Jamovi 2.2.5 (The jamovi project, www.jamovi.org, accessed on 1 March 2022). A multiple linear mixed model was used to assess differences across the three timepoints, with Holm post hoc tests identifying specific pairwise changes. The model included participant, timepoint, test type, and age as predictors, with test scores as outcome variables. Although the sample size was small (n = 7), a mixed model was chosen because it accommodates repeated measures within participants, enables covariate adjustment, and makes use of all available data. The results should be interpreted with caution, as the design and sample size limit statistical power and generalizability. Statistical significance was set at α = 0.05, with a critical F-value of 3.88. Motivation was assessed by averaging self-reported ratings across four training periods. Changes in reaction time were evaluated by comparing the average number of lights per second between the first and final training sessions.

## 3. Results

Of the 11 individuals invited, 7 completed the study. Three did not attend the initial assessment, and one withdrew after baseline due to rehabilitation admission. All completers attended at least 11 of 12 training sessions. One participant had a medication change during the study, and no serious adverse events were reported (Table 2).

**Figure 1:**
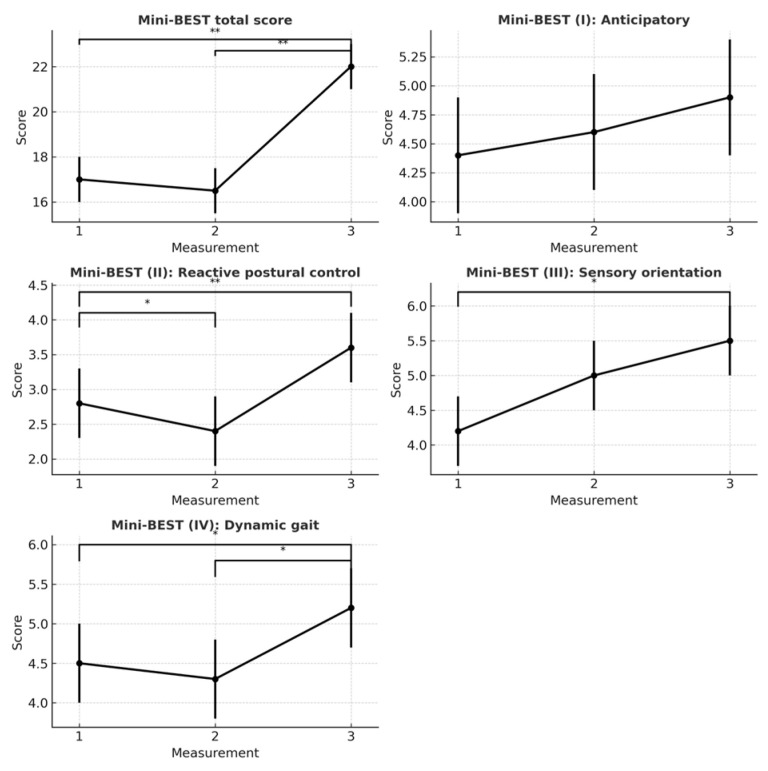
Mini-BESTest score. * *p* < 0.05 and ** *p* < 0.001 based on multiple linear mixed-model analysis.

Total Mini-BESTest scores increased significantly over time (F = 57, *p* < 0.001, R^2^ = 0.924), adjusted for age. Subcomponent analysis showed significant improvements in reactive postural control (Part II; F = 29.79, *p* < 0.001, R^2^ = 0.754), sensory orientation (Part III; F = 8.4, *p* = 0.005, R^2^ = 0.724), and dynamic gait (Part IV; F = 10.97, *p* = 0.002, R^2^ = 0.750). No significant change was observed in anticipatory postural control (Part I; F = 2.00, *p* = 0.179, R^2^ = 0.661); see Figure 1. Holm-adjusted post hoc tests showed significant differences between pre- and post-training (2–3) and baseline and post-training (1–3), with no difference between baseline and pre-training (1–2).

The 360° Turn Test showed a significant reduction in completion time over the intervention period (F = 7.98, *p* = 0.006, R^2^ = 0.749). No significant changes were found in Timed Up and Go (F = 3.00, *p* = 0.088, R^2^ = 0.778), 5× Sit to Stand (F = 1.49, *p* = 0.264, R^2^ = 0.569), or the 10 m walk test (F = 2.54, *p* = 0.120, R^2^ = 0.698).

Motivation ratings remained stable at 10/10 for four participants (1, 3, 6, and 7). Participant 2 rated the training 9.67 initially and 10 thereafter. Participant 5’s motivation increased across sessions. Participant 4’s ratings declined slightly after an initial average of 9.33. Average reaction time decreased by 0.75 ± 0.41 s per light across participants.

No adverse effects, such as falls, were observed throughout the training sessions.

## 4. Discussion

This study found that four weeks of agility training using a light-based system significantly improved balance in individuals with Parkinson’s disease. Notable improvements were observed in reactive postural control, sensory orientation, and dynamic gait, while anticipatory postural control remained unchanged. In terms of functional performance, participants demonstrated a significant improvement in turning ability, as measured by the 360° Turn Test. However, no significant changes were detected in transfer ability, walking speed, or the Timed Up and Go test. Regarding motivation, all participants reported the training as highly engaging, with motivation levels remaining stable in four participants, increasing in two, and slightly decreasing in one throughout the intervention period.

### 4.1. Effect on Balance

Postural instability is a major contributor to disability in Parkinson’s disease, as individuals struggle to maintain balance when their center of mass shifts beyond their base of support [35,36]. Sufficiently large and fast stepping responses, essential for fall prevention, are often impaired [37]. This study found that four weeks of agility training with a light-based system improved balance, particularly reactive postural control and dynamic gait—likely due to training large, rapid, multidirectional steps. These findings align with previous research showing that movement speed and amplitude can be improved in individuals with the akinetic–rigid subtype [38,39]. Sensory orientation also improved significantly. Overall, these findings are consistent with those of Okubo et al. [40], who reported that step training significantly reduced falls and improved balance and reactive control in older adults. Our training, as in previous studies, emphasized large, rapid, multidirectional steps—essential for feed-forward control and fall prevention. Improvements in reactive control and reaction time suggest this involved step reaction training.

### 4.2. Effect on Functional Performance

General functional mobility is essential in physiotherapy, and the TUG test is commonly used to monitor changes over time [41]. TUG scores did not improve significantly. While the lack of statistical significance may be due to the limited sample size, the observed trend indicates that meaningful effects might emerge with greater participation or an extended training period. This aligns with Garcia et al. [42], who found no change in TUG after 4 weeks of step training in older adults but noted improvements after 8 and 12 weeks. Similarly, Lai et al. [43] reported TUG improvements following a 6-week training program, with effects sustained after 6 weeks, sustained at follow-up. In this study, no significant changes were seen in transfer ability (5× Sit to Stand) or walking speed (10 m walk), but turning ability improved significantly. The circular elevated light setup may have favored rapid directional changes over deep lunging, squatting, or walking movements.

### 4.3. Effect on Motivation

Apathy affects about 40% of people with Parkinson’s disease across all stages and is marked by reduced motivation, emotion, and goal-directed behavior [11]. It is linked to diminished reward sensitivity, but external motivation and feedback may help activate reward pathways and encourage movement [44]. The light-based training system offered continuous performance feedback, making it well suited for individuals with Parkinson’s disease. Four of seven participants consistently rated motivation as 10/10, with most reporting increased motivation over time. High motivation was evident through strong attendance, engagement, and improvements in reaction time, likely driven by real-time feedback and the use of colorful lights. Feedback is known to enhance effort, enjoyment, and cognitive engagement [13]. However, it should be acknowledged that the consistently high motivation scores may have been influenced by the presence of the researcher during sessions, which could have inflated ratings. Similar findings were reported by Pompeu et al. [45] using Wii Fit. All participants noticed benefits post-intervention; five felt significantly faster and more agile in movement and thought, while two reported mild physical improvements.

### 4.4. Strengths and Limitations

A key strength of this study is that it is the first to examine light-based agility training in individuals with Parkinson’s disease. All participants had the akinetic–rigid subtype, characterized by pronounced bradykinesia, making them a well-defined and relevant target group. However, this study has several limitations. The small sample size (n = 7) limits statistical power, and a larger cohort would better clarify the training’s effects. Although mixed-model analysis allowed repeated measures to be fully utilized, the findings should be interpreted as exploratory rather than confirmatory. A larger, adequately powered study is needed to clarify the true magnitude and reliability of training effects. Generalizability is also restricted. The participants were narrowly defined—akinetic–rigid subtype, Hoehn and Yahr stages II–III, and under 81 years—which excludes other subtypes and disease stages. Therefore, the results cannot be assumed to apply to the broader PD population.

Additional limitations include the same researcher conducting both training and assessments, introducing potential bias despite adherence to standardized protocols. Assessment timing varied, with the baseline conducted later in the day, possibly affecting the results due to medication or fatigue, though no significant baseline–pre-differences were observed. Finally, motivation ratings were provided in the researcher’s presence, possibly introducing social desirability bias. While no validated reference exists for this approach, the use of simple numeric rating scales is common in clinical and rehabilitation settings, and the method was chosen for its feasibility.

## 5. Conclusions

A four-week light-based agility training program improved balance and turning ability in individuals with Parkinson’s disease, particularly enhancing reactive postural control, sensory orientation, and dynamic gait, although functional mobility outcomes showed limited change. The program was well tolerated and highly engaging, with motivation remaining consistently high or increasing for most participants. Larger controlled studies are needed to confirm these findings and to establish light-based agility training as a complementary rehabilitation approach.

## Figures and Tables

**Table 1:** Inclusion and exclusion criteria.

Inclusion Criteria	Exclusion Criteria
Hoehn and Yahr stage II–III	Other neurological disorders (e.g., stroke, multiple sclerosis)
Akinetic–rigid subtype confirmed by neurologist	Severe musculoskeletal disorders afffecting gait, balance, or rising from a chair
UPDRS thresholds: 1.1 Cognitive impairment: 0–2 (normal–mild) 2.12 Gait and balance: 1–2 (slight–mild) 2.13 Freezing of gait: 0–1 (normal–slight) 3.10 Gait: 1–3 (slight–moderate) 3.12 Postural stability: 1–2 (slight–moderate) 3.14 Global spontaneity of movement (bradykinesia): 2–4 (mild–severe)	Significant cardiovascular disease limiting safe participation Severe visual impairment interfering with mobility tasks History of brain surgery

Note: UPDRS item codes (e.g., 1.1 and 2.12) correspond to the Movement Disorder Society—Unified Parkinson’s Disease Rating Scale (MDS-UPDRS).

**Table 2:** Baseline characteristics of study participants (n = 7).

Variable	Mean (SD)	Min	Max	95% CI
Age (years)	74.9 (3.57)	69	81	73.3–76.4
Height (cm)	173 (7.63)	165	187	169–176
Weight (kg)	82.6 (4.98)	79	94	80.5–84.7
HY stage	2.86 (0.36)	2	3	2.7–3.01
Falls past year	1.00 (1.73)	0	5	0.26–1.74
Disease duration (years)	7.57 (2.50)	3	11	6.5–8.64
SGPALS	2.29 (0.90)	1	3	1.9–2.29

Abbreviations: HY = Hoehn and Yahr stage; SGPALS = Saltin–Grimby Physical Activity Level Scale.

## Data Availability

The dataset used and analyzed in this study is available from the corresponding author. The data are not publicly available because open access approval was not obtained from the National Bioethics Committee.
